# Ribosome Heterogeneity in Plants: The Causes of This Phenomenon and Its Implications on Gene Expression

**DOI:** 10.3390/plants15132043

**Published:** 2026-07-01

**Authors:** Qilin Zhen, Yongsheng Bai, Beixin Mo, Wei Xiong

**Affiliations:** Guangdong Provincial Key Laboratory for Plant Epigenetics, Longhua Bioindustry and Innovation Research Institute, College of Life Sciences and Oceanography, Shenzhen University, Shenzhen 518060, Chinabaiys_297@foxmail.com (Y.B.)

**Keywords:** ribosome, mRNA translation, ribosomal protein (RP), ribosomal DNA/RNA (*rDNA*/rRNA), heterogeneity

## Abstract

Ribosomes are essential macromolecular complexes responsible for protein synthesis and have traditionally been regarded as uniform and passive components of translational machinery. However, accumulating evidence has revealed that ribosomes exhibit substantial heterogeneity in both composition and function. In this review, we summarize the major sources of ribosome heterogeneity in plants, including ribosomal protein (RP) paralog diversity, sequence variation in *rDNA*/rRNA, dynamic chemical modifications of rRNAs and RPs, alterations in RP stoichiometry, and the involvement of ribosome-associated factors. These mechanisms collectively generate structurally and functionally distinct ribosome populations. Emerging evidence suggests that these heterogeneous ribosomes can actively regulate gene expression by preferentially translating specific subsets of mRNAs in response to developmental cues and environmental conditions. We further discuss the potential biological implications of ribosome heterogeneity in plant growth, development, and stress adaptation, and highlight current challenges in the field. Advances in high-resolution structural and single-ribosome profiling technologies are expected to provide new insights into the regulatory roles of heterogeneous ribosomes. This review provides a comprehensive framework for understanding the causes and functional significance of ribosome heterogeneity in plants, offering new perspectives on translational regulation and plant adaptive biology.

## 1. Introduction

Ribosomes are biomacromolecular assemblies responsible for protein synthesis, composed of ribosomal proteins (RPs) and RNAs (rRNAs). The high-resolution crystallographic studies of the ribosome conducted by the research groups of Ramakrishnan, Steitz, Yonath, Ban, and Yusupov have established the core structural theoretical framework in this field, providing a structural foundation for understanding how mRNA-carried genetic information is decoded and how polypeptides are synthesized [1,2,3,4]. Structurally, a single ribosome consists of two parts: the small ribosomal subunit (SSU) and the large ribosomal subunit (LSU). The prokaryotic 70S ribosome is assembled from 50S LSU and 30S SSU, while the eukaryotic cytosolic 80S ribosome is assembled from 60S LSU and 40S SSU [5,6]. In plants, the 60S LSU harbors 48 distinct RPs and 5S, 5.8S, and 25S rRNAs, while the 40S SSU is composed of 33 different RPs and the 18S rRNA [7]. Recently, the near-atomic resolution (2.2 Å) structure of the plant 80S ribosome in an actively translating state was determined from tobacco using cryo-electron microscopy, which further revealed the global architecture and functional characteristics of the plant cytoplasmic ribosome [8].

In eukaryotic cells, ribosome biogenesis exhibits distinct spatial compartmentalization and hundreds of ribosome biogenesis factors (RBFs) are involved in this complicated process [9,10]. The precursors of 18S, 5.8S and 28/25S rRNAs are co-transcribed from the ribosomal DNA (*rDNA*) loci by RNA polymerase I (Pol I) within the nucleolus, whereas the precursor of 5S rRNA is transcribed separately by polymerase III (Pol III) in the nucleoplasm [11]. With assistance of RBFs, pre-rRNAs undergo a series of cleavage and modification steps to generate mature rRNAs. RP-encoding mRNAs are translated in the cytoplasm and the resulting RPs are imported into the nucleus via nuclear pore complexes [12]. Once inside the nucleus, RPs bind to their processed rRNA partners, leading to the separate assembly of the pre-40S and pre-60S subunits. These pre-assembled ribosomal subunits are subsequently transported into the cytoplasm, where they undergo final maturation to become functional ribosomes.

In a translating ribosome, rRNAs serve as both the structural framework and the active center that catalyzes peptide bond formation [13]. Complementary to and mutually reinforcing the functions of rRNAs, RPs play vital roles in stabilizing the ribosomal structure, assisting in rRNA folding, and participating in translation factor binding [14]. In addition to their basic roles in translation, some RPs possess specific modulatory roles. For example, *Arabidopsis* RPS2B can form a complex with PRMT3 and cooperate with PDCD2 to participate in ribosome assembly and nucleocytoplasmic transport and balance plant growth and cold stress responses through regulating translation [15]. Each RP occupies a spatially defined position adjacent to core functional hubs including the peptidyl transferase center (PTC), mRNA entry channel, tRNA-binding pockets and polypeptide exit tunnel. Long disordered extensions of RPs bridge spatially distant functional modules, forming conserved inter-protein communication networks that mediate allosteric signal transmission across the ribosome cavity. Moreover, RPs possess long disordered extensions that can bridge spatially distant functional modules, forming a conserved inter-protein communication network [16].

The conventional process of protein translation in eukaryotic cells occurs in four stages: initiation, elongation, termination, and ribosome recycling. During initiation, the eIF2-Met-tRNAi-GTP ternary complex assembles with the 40S SSU, forming the 43S pre-initiation complex. This complex binds to the 5′ end of an mRNA and scans for the translation start codon, after which the 60S LSU joins the SSU, forming the active translating ribosome. During elongation, genetic information carried by mRNAs is decoded by the SSU, and aminoacyl-tRNA molecules sequentially deliver amino acids. Termination occurs when ribosomes encounter a stop codon, releasing the nascent polypeptide. The subsequent ribosome recycling process disassembles ribosomes for reuse [17]. Importantly, translation is precisely regulated by various cis-acting elements in mRNAs, trans-acting factors, and conserved signaling pathways, thereby affecting the efficiency and specificity of protein synthesis in plants in response to internal and external environmental signals [18,19].

Ribosomes were conventionally regarded as passive machines, translating mRNAs into proteins without sequence preferences. However, recent studies have revealed that ribosomes in the eukaryotic cells are not homogeneous: the existence of paralogs of RPs, variations in rRNAs, dynamic modifications of both of RPs and rRNAs, substoichiometry of RPs and the presence of unstably associated non-ribosomal proteins contribute to the production of distinct ribosome types. Studies in different species have demonstrated that heterogeneous ribosomes, with their unique components, have the ability to regulate gene expression through preferential translation of specific mRNAs [20,21,22,23].

In plants, ribosome heterogeneity is more pronounced than in other species due to the extensive duplication of RP-encoding genes [24,25,26], thereby increasing the diversity of subsets of ribosomes and significantly enhancing the potential for translation regulation. This article systematically reviews the current evidence that supports ribosome heterogeneity in plants and explores their verified and possible influences on modulation of gene expression, aiming to provide new perspectives and theoretical foundations for a deeper understanding of ribosome function.

## 2. Duplication of RP Genes

In different organisms, multiple genes can encode the same RP, and the existence of RP paralogs leads to ribosome diversity within species and even within a single cell. From an evolutionary perspective, the patterns of RP gene duplication differ significantly among different biological groups.

In bacteria, ribosomes, consisting of approximately 55 RPs and 3 rRNAs, are relatively smaller than their eukaryotic counterparts. Although most RP genes in bacteria present as a single copy, there are still a few exceptions. For instance, in *Escherichia coli*, at least two different RPs, namely the bL31 and bL36 RPs, have functional paralogs [27]. Additionally, some bacterial RP genes are often organized into operons, ensuring coordinated co-transcription and co-translation for efficient ribosome assembly [28]. Moreover, nearly half of the bacterial RP genes are concentrated in one or a few genomic loci rather than being scattered throughout the genome, allowing their expression to be coordinately regulated to accommodate rapid growth rates [29].

In yeast, most RPs have paralogs (59 RP families in *Saccharomyces cerevisiae* and 58 in Schizosaccharomyces pombe are duplicated). In *S. cerevisiae*, RP paralogs are mainly derived from whole-genome duplication (WGD) events, whereas *Sch. pombe* lacks WGD and RP genes are mainly duplicated through retrotransposition [30]. The core significance of the yeast RP gene multi-copy status lies in the formation of functionally specialized ribosomes through functional divergence of paralogous proteins, thereby enabling selective translation of specific mRNAs to adapt to different physiological states and stress conditions [31,32,33,34,35]. Meanwhile, dosage compensation effects mitigate the impact of mutations or expression suppression in individual copies to maintain ribosome homeostasis [36]. Furthermore, paralogs have acquired ancillary functions beyond their core assembly roles, ultimately achieving both the maintenance of translational homeostasis and functional diversification [34,37,38].

In mammals, most RPs are encoded by a single functional gene, while numerous nonfunctional RP pseudogenes are present in the genome, which are mainly generated by retrotransposition [39]. For instance, the human RPS4 family comprises three paralogs, RPS4X, RPS4Y1, and RPS4Y2, with RPS4Y2 being specifically expressed only in the testes [40,41,42].

Compared to the aforementioned organisms, plants exhibit the highest degree of RP gene duplication, driven primarily by WGD and tandem duplication—about 57% in *Arabidopsis* and 42% in rice from WGD, and 10% and 17% from tandem duplication, respectively [25]. Besides these two major duplication mechanisms, retrotransposition events also account for 1% and 2% of RP gene duplications in *Arabidopsis* and rice, respectively. Together, these mechanisms shape the extremely high multi-copy nature of RP genes in plants. In *Arabidopsis*, rice, tomato, and *Brassica napus*, nearly every RP has two or more functional paralogs, with sequence identity ranging from 40% to 100%.

Despite high sequence identity among paralogs, differences in their noncoding regulatory elements, such as promoters and untranslated regions (UTRs), may lead to differential expression patterns. These variations contribute to distinct expression of RP paralogs across different tissues, different developmental stages, and responses to environmental stresses [22,24]. Due to the higher degree of duplication of RP coding genes in plants, ribosomes in plants are more heterogenous than their counterparts in other species, increasing the possibility that the same or different mRNAs may be translated by distinct ribosomes with differential efficiencies in plants.

In *Brassica napus*, transcript profiling of RP genes using EST data revealed that a large fraction of RP genes were differentially expressed and expression patterns of RP paralogs varied extensively across tissue types, suggesting that paralogous RPs probably have divergent functions and implying the engagement of specialized ribosomes in different growth and developmental stages and tissue-specific processes [24]. In *Arabidopsis*, the expression patterns of paralogous RP genes fall into two main categories: coordinated expression and specialized expression. In most RP families, RP paralogs exhibit clearly synchronized expression dynamics. For example, members of the EL34 (RPL34), US14 (RPS29) and UL23 (RPL23a) families show concurrent expression changes across different tissues and developmental stages, differing only in their expression levels [43,44]. This coordinated expression pattern is contributed to by shared regulatory elements, ensuring the dosage balance of ribosomal proteins required for ribosome assembly [45,46]. In contrast, some paralogous RP genes, like those in the US19 (RPS15) family, exhibit strict spatiotemporal specificity: US19U (RPS15A) and US19X (RPS15D) are broadly expressed, whereas US19Z (RPS15B) and US19Y (RPS15C) are expressed only in developing seeds and floral organs [43]. Interestingly, paralogous genes for some RP families were found to respond differently to stresses; for instance, the UL16 (RPL10) family paralogs exhibited markedly distinct responses to UV-B stress: UL16Z (RPL10A) remained unaffected by UV-B stress, UL16Y (RPL10B) was downregulated, whereas UL16X (RPL10C) was significantly upregulated [47]. Altogether, organ-specific, developmental stage-specific, and stress-induced expression of paralogous RP genes endow ribosomes in different tissues or developmental phases with distinct ribosomal protein compositions which may lead to functionally specialized ribosomes.

Mutations of paralogous RPs do not always lead to the same phenotypic outcomes. In *Arabidopsis*, the UL18 (RPL5) family contains two members, namely UL18Z (RPL5A) and UL18Y (RPL5B), and inactivation of either leads to pleiotropic developmental defects [48,49]. In contrast, the UL23 (RPL23a) and UL3 (RPL3) RP families, each containing two family members, exhibit different patterns. Null mutants of UL23Y (RPL23aB) and UL3Y (RPL3B) show phenotypes nearly identical to the wild type, whereas down-regulation of UL23Z (RPL23aA) and UL3Z (RPL3A) results in obvious abnormalities, with null mutants being embryo-lethal [44,50]. Despite these paralogous RPs having different phenotypes, genetic experiments have demonstrated that they are functionally interchangeable and exhibit dosage effects. Their different phenotype differences are actually caused by their different expression levels, and paralogs with high expression levels have a greater impact on phenotypic outcomes. Until now only a limited number of RP families have been experimentally investigated to validate their functions; the functional divergences between paralogous RPs in plants need further exploration.

Although the core functions of ribosomes typically remain unchanged, sequence variations in these RP isoforms may change surface charge distribution or spatial conformation, thereby affecting the efficiency of ribosomes to recognize and bind regulatory elements such as specific mRNA 5′UTR or IRES. Such heterogeneity of ribosomes may allow specialized ribosomes to selectively prioritize the translation of distinct mRNAs, thereby precisely regulating gene expression.

## 3. Sequence Variation in rDNAs

In addition to the ribosome heterogeneity resulting from RP paralog switches, variations in rRNA coding sequences (*rDNAs*) may also lead to ribosome heterogeneity. The arrangement of *rDNAs* in eukaryotic and prokaryotic organisms exhibits distinct structural characteristics: *rDNAs* in eukaryotes are intensively organized into the nucleolus organizer regions (NORs) on specific chromosomes, whereas prokaryotes lack such substructures [51]. In bacteria, *rDNAs* are primarily arranged as tandem repeats of the 16S-23S-5S operon, which are usually present in multiple copies and in a dispersed distribution across different chromosomal loci [52]. In yeast, *rDNAs* are mainly concentrated into the NORs on chromosome XII, existing as a multi-copy tandem repeat cluster [53,54]. Each repetitive unit contains the 35S pre-rRNA coding sequence (precursor of the 18S, 5.8S, and 25S mature rRNAs) [55]. In animals, the 18S, 28S, and 5.8S rRNAs are co-transcribed as a single precursor rRNA from the *rDNA* loci, the organization of which exhibits a typical multi-chromosomal distribution pattern, with multiple NORs located on the short arms or satellite regions of these chromosomes. Each NOR contains hundreds of tandemly repetitive *rDNA* transcription units [56,57,58].

In plants, the 45S *rDNA* genes are also arranged as hundreds of tandemly repetitive units, forming NORs adjacent to the heterochromatic regions. The *Arabidopsis* NORs are located on chromosomes 2 and 4, respectively known as NOR2 and NOR4 [59,60]. Each repetitive unit contains the 45S *rDNA* gene, encoding the precursor for the 18S, 5.8S, and 25S mature rRNAs, which are co-transcribed by the RNA polymerase I (Pol I) [61,62,63]. The promoter of each 45S *rDNA* gene typically contains core functional areas and upstream regulatory elements, which specifically recruit Pol I and related transcription factors. The transcribed 45S pre-RNA is rapidly processed into the 35S pre-rRNA. Structurally, the 35S pre-rRNA contains two external transcribed spacers (5′-ETS and 3′-ETS), and the coding sequences correspond to the 18S, 5.8S, and 25S mature rRNAs, which are separated by two internal transcribed spacers (ITS1 and ITS2). Through a series of endo- and exonucleolytic cleavages, the spacer sequences are progressively removed, ultimately releasing the three mature rRNAs [61,64,65].

Adjacent 45S *rDNA* repeat units are separated by the intergenic spacer (IGS). This region not only physically delimits individual repeat units, but also typically contains species-specific repetitive sequences, *rDNA* gene promoters, transcription terminators, and signal sequences involved in pre-rRNA processing, thereby participating in the regulation of *rDNA* transcription efficiency [66,67,68]. As described above, plant genomes harbor hundreds of repetitive *rDNA* units; these repetitive *rDNA* units also exhibit significant sequence polymorphism, which leads to the transcription of a variety of divergent rRNA precursors from them. Sequence variations among *rDNA* units are distributed with obviously structural biases. Non-transcription regions such as the IGSs, and transcribed sequences that are excluded from the mature rRNAs, including the 5′ and 3′ ETSs and the ITSs, are hotspots for variation. Because these regions do not contribute directly to the structure of mature ribosomes, they are subject to relatively relaxed evolutionary constraints. Based on these variations, *rDNA* units are generally divided into several different subtypes. Among these variable regions, the IGS exhibits the highest level of variability, largely driven by unequal homologous recombination events, and is used as a key criterion for *rDNA* subtype classification. Differences in IGS sequences exist not only between species, but also between different populations of the same species, and even among individual *rDNA* units within a single genome. Species-specific repetitive elements and transcriptional regulatory elements are located in the IGS regions; variations in these regions may directly lead to distinct transcriptional activities of different *rDNA* subtypes, thereby contributing to ribosome heterogeneity [66,69,70].

Compared to the extensive variations in the IGSs, the 5′ and 3′ ETSs also exhibit notable but relatively lower levels of sequence and structural variation [71]. Sequence variations in the 3′ ETS region may affect its recognition by RNase III-type enzymes such as RTL2, thereby interfering with the co-transcriptional cleavage of pre-rRNAs [72]. Whereas the 5′ ETS serves as the initiation region for co-transcriptional processing of pre-rRNA, its core processing sites are relatively conserved in sequence to ensure the basal processing function required for pre-rRNA maturation, with sequence polymorphism, is often enriched in non-core regions [73,74]. In contrast, the ITS1 and ITS2 regions carry chromosome-specific signatures. In *Arabidopsis*, ITS1 variation is manifested by the presence or absence of an AvaI restriction enzyme recognition site [75], while ITS2 variation is characterized by insertions or deletions of the CAT trinucleotides. Notably, the CAT insertion in ITS2 frequently co-occurs with the AvaI site in the ITS1, serving as a hallmark of NOR2; in contrast, *rDNA* units lacking the CAT insertion are predominantly distributed on NOR4 [75]. In summary, sequence variations in the non-transcribed regions of *rDNA* units influence *rDNA* transcriptional activity and alter pre-rRNA processing rates, leading to differential rRNA synthesis and thereby promoting ribosome heterogeneity in plants.

Following transcription in the nucleolus, 45S pre-rRNAs are progressively processed into mature rRNAs by several endonucleases and exonucleases with the assistance of hundreds of ribosome biogenesis factors (RBFs). Concurrent with pre-rRNA maturation processes, the 18S rRNA is assembled into the small ribosomal subunit (SSU), whereas the 25S and 5.8S rRNAs are incorporated into the LSU. Although the sequences of mature rRNAs are highly conserved across different *rDNA* variants, they still exhibit limited chromosome-specific sequence polymorphisms [70]. rRNAs serve as both the catalytic core and structural scaffold of the ribosome and are essential for mRNA translation. The sequence conservation of mature rRNAs ensures the structural and functional stability of ribosomes, while limited sequence polymorphisms provide a potential source for the generation of heterogenous ribosomes.

In *Arabidopsis*, 18S *rDNAs* exhibit single-nucleotide polymorphisms (SNPs) and insertions/deletions (indels), and these variants are mainly concentrated in repetitive *rDNA* units on NOR2, serving as important markers for NOR2-specific *rDNAs* [75]. These SNPs and indels lead to sequence differences between the 18S rRNAs transcribed from NOR2 and those from NOR4, representing an important source of rRNA sequence heterogeneity. In contrast, 25S *rDNAs* only exhibit SNP variations without significant indels, and the polymorphic sites are mainly distributed among repetitive *rDNA* units on NOR4 [62,75]. Studies have shown that, in translating ribosomes, 25S rRNA variants are stably detectable and exhibit tissue-specific expression patterns [75], which further supports the existence of tissue-specific specialized ribosomes.

The sequences corresponding to 5.8S rRNAs are the most conserved regions among mature rRNA coding sequences, exhibiting almost no variations; this extreme conservation likely stems from its critical role in ribosome structure and function. Specifically, in plant ribosomes, 5.8S rRNA is an indispensable component of the 60S large subunit. It interacts with core ribosomal proteins near the peptide exit tunnel, participates in ribosome assembly and nascent chain sensing, and is closely associated with translational fidelity, efficiency, and adaptation to environmental stress [76,77]. Therefore, any alteration in 5.8S rRNA sequence would most likely disrupt this key molecular interaction, impair ribosomal function, and consequently reduce organismal fitness. This strong functional constraint drives the purging of deleterious variants by natural selection, explaining the extreme evolutionary conservation of 5.8S rRNA.

Although only the mature rRNAs, which exhibit limited sequence variations, are incorporated into the ribosomes, different *rDNA* variants may undergo differential transcription, producing pre-rRNA transcripts that are subsequently processed in distinct pathways to accommodate the growth and developmental stages and the changing environment. As a matter of course, these limited sequence variations in mature rRNAs could directly lead to the formation of heterogenous ribosomes in plants. In *E. coli*, sequence variations in mature rRNA have been shown to affect ribosome functional properties, alter tRNA accommodation and entry efficiency under tetracycline, and regulate the expression of specific stress-response gene sets [78]. In plants, distinct ribosome populations have been identified in leaves, inflorescences and siliques, which arise from the incorporation of rRNAs transcribed from different *rDNA* variants [75]. These findings provide solid experimental evidence for the existence of heterogenous ribosomes contributed by variations in *rDNAs*. However, whether these heterogenous ribosomes arising from variations in mature rRNAs have functional discrepancies remains to be investigated in plants.

## 4. Dynamic Modifications of rRNAs and RPs

The ribosome is a complex macromolecular machine composed of rRNAs and RPs, whose structure, function, and translational efficiency are significantly modulated by modifications on both rRNAs and RPs. RPs are anchored to the periphery of, or are in direct contact with, the core functional hubs of translation, including the peptidyl transferase center (PTC), the polypeptide exit channel, the mRNA entry channel, and the A/P/E-site tRNA-binding pockets. Within these key microdomains, RPs perform two major functions: stabilizing tRNAs, mRNAs, and translation cofactors, and mediating long-distance molecular signaling between spatially separated functional modules of the ribosome. The vast majority of eukaryotic RPs possess disordered N-terminal or C-terminal extensions that traverse the internal cavities of the ribosome, forming an extensive inter-protein communication network [16].

Recent studies have shown that the complexity of the inter-protein communication network increases progressively during evolution, and that this network serves as a core coordinating element for the global conformational dynamics during ribosomal translocation [16]. In the process of conformational regulation, the electrostatic distributions of RPs and rRNAs play a critical role: local electrostatic changes can affect their interactions and be transmitted over long distances through the protein interaction network, ultimately driving global conformational transitions of the ribosome [79]. It can thus be inferred that modifications such as acetylation, phosphorylation, and pseudouridylation, by altering the surface electrostatic distributions of RPs and rRNAs, or by modulating the interaction properties between amino acid residues, may influence the RP interaction network, impede signal transmission between distal domains of the ribosome, remodel ribosomal conformational dynamics, and ultimately produce differential translation products, thereby giving rise to translational heterogeneity.

The modifications on rRNAs are incorporated during their maturation, with the most prevalent types being methylation and pseudouridylation, mainly guided by specific small nucleolar ribonucleoprotein (snoRNP) complexes [80,81,82]. In plants, 2′-O-ribose methylation of rRNA is mediated by C/D box snoRNPs, whereas pseudouridylation of rRNA is conducted by H/ACA box snoRNPs. Additionally, rRNAs also undergo specific base modifications such as m^6^A and m^5^C, which are mediated by dedicated modification enzymes including METTL5 and NSUN5 [12,83,84,85]. These modifications affect the folding and processing of the pre-rRNAs, thereby influencing the assembly of ribosomal subunits. Moreover, some specific modifications directly influence the translation accuracy, efficiency, and selectivity of ribosomes toward specific mRNAs by modulating the three-dimensional conformation of rRNAs [86]; the major modifications on rRNAs are summarized in Table 1.

Modifications on rRNAs are enriched in functionally critical regions of the ribosome. Specific modifications located within the decoding center have been proven to optimize the mRNA codon–tRNA anticodon pairing geometry, which improves translation fidelity. A highly conserved core structure, referred to as the “common core”, is shared across prokaryotes, archaea, and eukaryotes. This core encompasses the peptidyl transferase center (PTC) and nascent polypeptide exit tunnel (NPET) of the large subunit, as well as the decoding center (DC) of the small subunit [8]. In addition, the A, P, and E sites located at the interface between the small and large subunits, the tRNA translocation path, and the GTPase-associated region are also part of this core structure. Notably, regions of rRNA enriched in nucleotide modifications largely overlap with the “common core” [82]. This not only underscores the critical role of these modifications in ribosomal function but also reflects their strong evolutionary conservation. Such rRNA modifications may alter the local microenvironment of the inter-protein communication network surrounding the ribosomal functional core, indirectly interfering with allosteric signaling pathways between functional domains. Furthermore, different tissues harbor distinct rRNA modification profiles, giving rise to ribosomes with heterogeneous modification patterns [87].

Strikingly, rRNA 2′-O-methylation has undergone significant evolutionary changes, with the number of 2′-O-methylated nucleotides increasing along the phylogenetic tree, and the primary catalytic mechanism shifting from site-specific methyltransferases to the C/D box snoRNP complex. Furthermore, rRNA modifications are dynamically regulated rather than statically fixed: they are remodeled to accommodate developmental stage and organ morphogenesis, resulting in modification patterns that are both dynamic and spatiotemporally specific. For example, in plants, some methylation sites exhibit stage-specific patterns during growth [85]. Similarly, during stem development in *Arabidopsis*, thermospermine regulates the translation of SAC51-family mRNAs by modulating rRNA pseudouridylation and multiple RNA processing and modification processes, thereby controlling xylem differentiation and affecting stem elongation [88]. Furthermore, under environmental stresses such as heat shock, low temperature, and drought, cells rapidly alter the modification levels of specific rRNA sites, thereby directing the ribosome population to preferentially translate stress-responsive mRNAs. It has been reported that under low-temperature induction, OsPUS1 accumulates and catalyzes site-specific pseudouridine (Ψ) modifications of rRNA, thereby dynamically regulating rRNA processing, ribosome biogenesis, and translation efficiency in chloroplasts to maintain chloroplast function and cellular homeostasis under cold conditions [89].

In parallel, RPs are also subject to a variety of post-translational modifications, such as phosphorylation, acetylation and ubiquitination, which are dynamically regulated [45]. This dynamic nature of post-translational modifications on RPs is primarily achieved through the antagonistic action of modifying enzymes and de-modifying enzymes (main enzymes involved in RP modification and de-modification are summarized in Table 2). For example, phosphorylation is catalyzed by specific kinases and reversed by phosphatases [90]; acetylation is regulated by the balance between acetyltransferases and deacetylases [91]; and ubiquitination is catalyzed by ubiquitin ligases and reversed by deubiquitinating enzymes [92]. In the *Arabidopsis* immune signaling pathway, this reversible regulatory mechanism enables the modification status of specific RPs to rapidly respond to upstream MAMP signals, thereby achieving precise control of translation [90]. Changes in intracellular nutrient status, energy levels, or environmental conditions can be perceived through signaling pathways such as TOR. The TOR pathway dynamically regulates the modification status of the ribosomal protein RPS6 by modulating the activity of downstream kinases [93,94,95].

The modification state of RPs may influence their stability, subcellular localization, and protein–protein interaction patterns. For example, phosphorylation of certain RPs can regulate their nucleocytoplasmic transport [96]. Dynamic acetylation can alter the surface charge of RPs to regulate their stability, affect ribosome assembly and translation efficiency, and mediate metabolic signals and stress responses. Recent structural biology studies have shown that the electrostatic distribution patterns of RPs act as molecular switches, relying on an ‘electrostatic domino’ effect within the RP network [79]. Through periodic electrostatic repulsion/attraction between charged protein residues and rRNAs (CMs, the major Centers of Motion), they modulate relative RNA–protein displacements and drive global conformational dynamics of the ribosome. Moreover, modifications such as acetylation can change the charge distribution characteristics along the extended peptide regions of RPs, leading to alterations in the conserved allosteric signaling pathways between distant functional sites. Ultimately, this results in stable differences in the overall conformational dynamics of the ribosome, giving rise to structural and functional heterogeneity. In rice, its deacetylation is controlled by the cytoplasmic histone deacetylase HDA714. When ribosomes are hyperacetylated, their stability decreases and ribosome stalling occurs, indicating that the balance between acetylation and deacetylation is linked to translation efficiency and stress responses [97]. The ubiquitination level of RPs changes dynamically with physiological status, thereby regulating ribosome function. During development, it modulates ribosome activity and influences translational reprogramming [98,99]. During senescence, it promotes the degradation of redundant ribosomal proteins and reduces translational capacity [98,99,100]. In eukaryotes, ubiquitination may also influence the binding affinity of RPs for ribosomal subunits or translation-related factors by altering RP conformation [101], although direct evidence in plants is currently lacking.

These dynamic modifications of RPs could precisely tune ribosome assembly, translation initiation and elongation processes, and translational fidelity, thereby enabling cells to rapidly adapt to fluctuations in environmental signals and maintain intracellular proteome homeostasis [90,102,103]. For example, the *Arabidopsis* small-subunit ribosomal protein ES6 (RPS6) is a canonical target of TOR signaling, and phosphorylation at its Ser240 site serves as an indirect readout of TOR pathway activity. Under normal conditions, TOR activation drives high-level phosphorylation of ES6 Ser240 to sustain root meristem growth and cell proliferation. In contrast, under ABA stress, inhibition of TOR activity leads to reduced phosphorylation of ES6 Ser240 and suppression of root meristem activity, thereby contributing to stress adaptation [104].

Altogether, dynamic modifications of both rRNA and RP form additional regulatory layers that fine-tune ribosome function. Meanwhile, dynamic variations in the modification profiles of rRNAs and RPs alter their surface electrostatic landscapes, which disrupt long-range allosteric signaling pathways between ribosomal functional modules. These structural disturbances further amplify ribosome heterogeneity and endow ribosomes with sophisticated translational regulatory capacity, adjusting their activity and function in real time to respond to different cellular states and environmental changes.

**Table 1:** Main modifications on rRNAs.

Types	Enzymes/Complexes	Core Functions	References
2′-O-ribose methylation	C/D box snoRNP	It impacts pre-rRNA folding/assembly and influences ribosome assembly, translation accuracy, efficiency, and mRNA selectivity.	[12,82,85]
Pseudouridylation	H/ACA box snoRNP	It stabilizes rRNA functional domains, regulates fidelity, and dynamically remodels ribosomes during development (e.g., stem elongation) and stress.	[82,88,102]
m^6^A	METTL5	It can maintain ribosome structure and function and affect translation efficiency and light-signaling responses.	[84]
m^5^C	NSUN5	It can regulate rRNA processing and maturation, as well as translation fidelity.	[83]

**Table 2:** Post-translational modifications on RPs.

Types	Modification and De-Modification Enzymes	Core Functions	References
Phosphorylation	Kinases/Phosphatases	It can respond to internal and external signals, reversibly regulate the translation process and the intracellular localization of RPs, and mediate growth and stress adaptation.	[90,93,96,104]
Acetylation	Acetyltransferases/Deacetylases	It can alter the surface charge of RPs to regulate their stability, affect ribosome assembly and translation efficiency, and mediate metabolic signals and stress responses.	[91,97]
Ubiquitination	Ubiquitin ligases/Deubiquitinases	It can serve as a recognition signal for selective autophagy, mediating the wholesale degradation of defective or redundant ribosomes, and participates in ribosome quality control and stress responses.	[92,98,99,100]

## 5. Alterations in RP Stoichiometry

The classical ribosome assembly model dictates a strict 1:1 stoichiometry for most RPs except for the acidic ones, which are present in two copies within a single ribosome. However, recent evidence has revealed the existence of cellular populations of “incomplete” or substoichiometric ribosomes, characterized by missing specific RPs [22,105]. These ribosomes are not necessarily the defective products resulting from impaired ribosome biogenesis; on the contrary, they possess specific physiological functions by selectively modulating the translation of specific subsets of mRNAs. Thus, alterations in RP stoichiometry constitute another crucial dimension of ribosome heterogeneity.

Currently, differences in RP stoichiometry among ribosomes from different sources are primarily determined using quantitative mass spectrometry [106]. By measuring the absolute abundance of 15 core RPs in the translating polysomes from mouse embryonic stem cells, one study revealed that four of these RPs were significantly present in substoichiometric levels compared to others [21]. This finding directly confirmed the existence of translationally active ribosomes lacking one or more core RPs. Quantitative analysis of ribosomes from barley root tips has also confirmed that multiple core RPs exist at substoichiometric levels, further demonstrating that the stoichiometry of RPs is not constant in plant ribosomes [107].

Compared to other forms of ribosome heterogeneity, alterations in RP stoichiometry can directly lead to marked alterations in a ribosome’s three-dimensional structure and surface properties. Studies have shown that the absence of specific RPs may affect binding to translation initiation factors by altering the conformation of the mRNA channel [31,108,109]. These structural changes can lead to functional alterations, allowing heterogeneous ribosome populations to preferentially translate specific subsets of mRNAs, and thereby exert specific regulatory control over gene expression. For example, knockout of EL24Y (RPL24B) affects translation reinitiation of mRNAs containing an upstream open reading frame (uORF), thereby regulating the expression of auxin pathway-related genes in *Arabidopsis* [110]. In tobacco, some specific RPs could indirectly alter the conformation of mRNA-binding regions by influencing interactions involving extended segments on the ribosome surface [8].

Furthermore, studies in animals have uncovered that differential expression of specific RPs could drive the formation of specialized ribosome subsets with a translation bias towards cancer-promoting mRNAs, thereby fostering a growth advantage in neoplastic cells during tumor development [111]. Although most related studies have been conducted in animals, largely due to the reliance on quantitative mass spectrometry—a method that is technically challenging to apply to plant samples—only a few examples have been reported in plants, as discussed above. Nevertheless, it can be assumed that functionally specialized ribosomes with stoichiometric variation in RPs may be widespread in plants.

## 6. Non-Stable Ribosome-Associated Factors

Ribosome-associated non-ribosomal factors are a class of proteins that can physically interact with ribosomes but are not core components of the ribosome. Apart from the permanent RPs and rRNAs, these factors constitute the temporary components of the ribosome. Different types of ribosome-associated non-ribosomal factors can influence the translation selectivity of ribosomes through their dynamic binding and regulatory mechanisms, and thus serve as an important source of functional heterogeneity in ribosomes.

Plant ribosomes harbor a diverse array of ribosome-associated factors, which can be categorized into three major classes based on their functional characteristics: (1) translation-related factors; (2) ribosome-regulatory proteins; and (3) post-translational modification-associated factors (Table 3). Translation-related factors are the most extensively studied class, including eukaryotic initiation factors (eIFs), elongation factors (eEFs), and release factors (eRFs) [103]. Translation initiation factors in plants frequently occur as multiple gene copies and isoforms, which contribute to stress-responsive translational regulation [22,45,112,113]. These isoforms exhibit distinct spatiotemporal expression patterns and stress responsiveness. For example, the *Arabidopsis* eIF4E family comprises multiple isoforms that are differentially expressed in vegetative and reproductive organs, and are induced under stress conditions such as low temperature [114,115]. Upon binding to ribosomes, different isoforms specifically regulate the translation of distinct mRNAs, directing ribosomes to preferentially synthesize proteins associated with development or stress adaptation, thereby meeting growth demands [115]. Post-translational modifications of eEF-1A—including phosphorylation and phosphorglycerylethanolamine (PGE) modification—may contribute to the regulation of its cellular localization and molecular function [116]. In *Arabidopsis*, the release factors eRF1 and eRF3 coordinately participate in translation termination process, ensuring its accuracy and efficiency, thereby sustaining normal plant growth and development. Among these, eRF1 serves as the core factor in translation termination; it recognizes all three stop codons and, upon binding to the ribosome, catalyzes the hydrolysis of the peptidyl-tRNA ester bond through its conserved GGQ motif, thereby releasing the nascent polypeptide chain [117,118,119]. eRF3 is a ribosome- and eRF1-dependent GTPase. Through GTP hydrolysis, eRF3 participates in eukaryotic translation termination, and this hydrolysis is an essential step for rapid peptidyl-tRNA hydrolysis. Furthermore, eRF3 interacts with the C-terminal region of eRF1, jointly triggering conformational and structural rearrangements of eRF1 and the pre-termination ribosome complex, thereby synergistically enhancing translation termination efficiency [120]. Different release factors act in concert with distinct roles; the specific molecular mechanisms governing their critical regulation in plants remain to be further explored.

Ribosome-regulatory proteins represent another important class of ribosome-associated factors. Unlike translation-related factors, these factors do not directly participate in the translation process but primarily regulate ribosome stability and functional specificity through their dynamic binding to ribosomes. For example, in *Arabidopsis*, the unconventional G protein AtYchF1 regulates ribosome functional specificity through dynamically interacting with small-subunit proteins, thereby specifically modulating the translation of stress-related proteins [121]. This mechanism enables the plant to form functionally specialized ribosome subpopulations under salt stress, achieving a resource balance between growth and stress responses. Through the action of these binding factors, plants are able to rapidly adjust ribosome function in response to environmental changes, thereby enhancing their adaptability.

Post-translational modification-associated factors also constitute an important category of ribosome-binding factors. These factors, such as kinases, methyltransferases, and ubiquitin ligases, modify ribosome components or associated factors through post-translational modifications, altering their binding properties and functions, thereby driving the formation of heterogenous ribosomes with specialized functions. For example, under pathogen stress, the kinase GCN2 phosphorylates the initiation factor eIF2α, altering its function in translation initiation and thereby modulating the translational bias of ribosomes toward preferential translation of stress-responsive genes [122]. As a rapid regulatory mechanism, post-translational modifications of both stable and transient ribosomal components, mediated by these ribosome-associated factors, enable plants to dynamically adjust ribosome function in response to fast-changing environmental conditions.

Ribosome-associated factors shape various subsets of heterogeneous ribosomes in plants through three synergistic mechanisms: spatiotemporally regulated expression of the factors, dynamic ribosome binding, and post-translational modifications [22]. Differential expression patterns serve as the foundation for ribosome heterogeneity formation. Specifically, the expression of distinct ribosome-associated factors across different tissues, developmental stages, or stress conditions enables the enrichment of particular ribosome subpopulations in corresponding scenarios. Ribosome-associated factors dynamically bind to ribosomes, enabling rapid transitions between functional states, while post-translational modifications further refine functional differences among ribosome subpopulations by modulating the binding affinity of these factors, thereby ensuring precision in translational regulation [22,123].

The dynamic associations of ribosomes with different kinds of non-ribosomal factors exaggerate the heterogeneity degree of ribosomes; such an association is crucial for mRNA translation and is thereby critical for plant growth, development, and environmental adaptation. It is anticipated that additional non-ribosomal factors will be identified and their roles in translational regulation will be clarified in the future.

## 7. Future Perspectives

**Figure 1:**
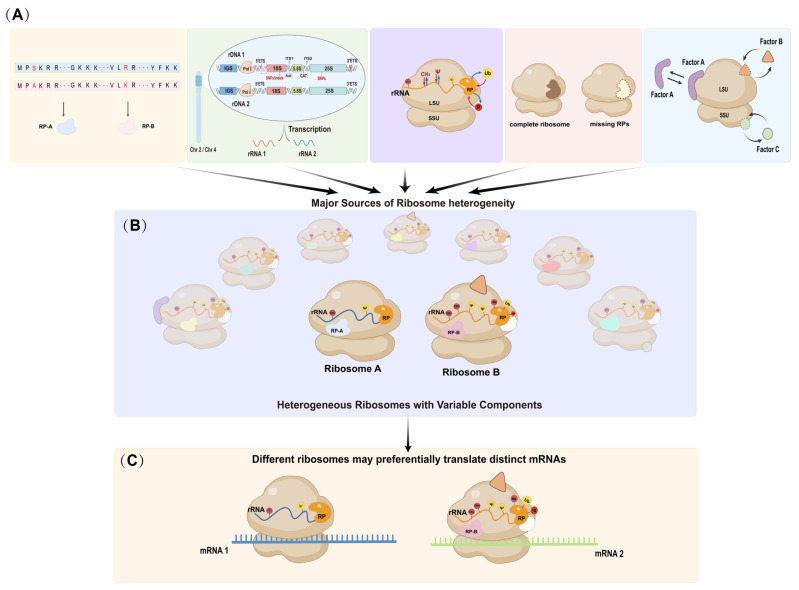
Factors that lead to ribosome heterogeneity in plants and the potential of heterogeneous ribosomes for selective mRNA translation. (**A**) Five core sources of ribosome heterogeneity in plants (from left to right): (1) paralogous RPs with similar but not identical amino acid sequences; (2) hundreds of repetitive *rDNAs* with sequence variations, generating different rRNA variants; (3) dynamic covalent modifications of rRNA (e.g., 2′-O-methylation and pseudouridylation) and RPs (e.g., phosphorylation and ubiquitination); (4) alterations in RP stoichiometry: omission of specific RPs in a fraction of ribosomes; (5) dynamically and reversibly associated non-ribosomal factors. (**B**) Plant ribosomes exhibit substantial heterogeneity in their composition contributed to by these abovementioned factors. (**C**) Different subsets of ribosome populations with specific components may have distinct functions via preferential translation of mRNAs. Heterogeneous ribosomes may selectively translate different mRNAs, allowing plants to differentially translate functionally distinct mRNAs, thereby achieving precise regulation of gene expression to adapt to the demands of different environments and developmental stages.

The components of distinct ribosomes within a single plant, and even in a single cell, may vary from one another, thereby rendering ribosomes dynamic, heterogeneous, and multifunctional regulators, rather than merely passive translation machines. This emerging field of ribosome heterogeneity (Figure 1) offers considerable opportunities for investigation, especially in plant systems. Currently, the primary challenge of this fascinating field is to delineate the complete landscape of heterogeneous ribosomes and to link specific compositional features to their distinct functions. Technological innovation is critically important to achieve this challenging goal. In particular, the integration of single-ribosome analysis approaches with increasingly advanced cryo-electron microscopy (cryo-EM) is crucial for classifying ribosome subpopulations across diverse cell types, developmental stages, and stress conditions.

Besides single-ribosome characterization, a deeper mechanistic understanding of the specialized functions of distinct ribosomes is also imperative. Key questions include: (1) How do cis-regulatory elements in mRNAs, such as specific IRES structures, upstream open reading frames, or codon usage bias, achieve precise recognition and preferential translation through interactions with specific ribosomes? (2) What are the intrinsic connections among RP paralog switching, *rDNA* variant shifts, modification pattern remodeling, addition or removal of specific RPs, and the binding or dissociation of non-ribosomal factors? (3) How is the spatiotemporal specificity of a ribosome subset determined? In addition, alternative splicing of messenger RNA can generate an array of mRNA isoforms that play important roles in regulating plant development and stress responses [124,125], and heterogeneous ribosome-mediated preferential translation offers great potential for elucidating the underlying mechanisms. Altogether, elucidating the mechanism by which heterogeneous ribosomes modulate gene regulation in plants holds significant potential for understanding environmental adaptation and for improving crop performance.

## 8. Conclusions

For a long time, the ribosome was regarded as a passive molecular machine solely responsible for protein synthesis. This review integrates multifaceted and compelling evidence that reshapes this perspective, revealing the ribosome as an active regulator of gene expression, and elaborating on the multiple layers of ribosome heterogeneity (as presented in the cartoon-style illustration in Figure 1). Diversity in ribosomal composition confers functional specificity, enabling distinct ribosome subpopulations to regulate gene expression through the preferential translation of specific mRNAs. Consequently, heterogeneous ribosomes exert precise control over development, differentiation, and stress adaptation.

In summary, the ribosome should be recognized as an additional regulatory hub, far surpassing its traditional role as a mere translation machine. Its inherent heterogeneity enables cells to achieve remarkable precision and adaptability in regulating their fates. Ongoing research into the structural basis of ribosome heterogeneity and its regulatory networks holds promise not only for deepening our understanding of fundamental biology but also for unveiling novel entry points to enhance crop resilience and productivity.

## Figures and Tables

**Table 3:** Classification and core functions of non-permanent ribosome-associated factors.

Ribosome-Associated Factors	Classification	Core Functional Effects	References
Translation-related factors	Eukaryotic initiation factors (eIFs)	It regulates translation initiation of specific mRNAs, while distinct isoforms guide ribosomes to preferentially translate development- or stress-related genes under corresponding developmental or stress conditions.	[22,115]
	Elongation factors (eEFs)	Elongation factors such as eEF-1A modulate translation elongation rate in response to cellular metabolic status and stress signals.	[22,116]
	Release factors (eRFs)	It ensures accurate/efficient translation termination for normal growth and development.	[117,118,119,120]
Ribosome-regulatory proteins	Unconventional G protein	It dynamically binds ribosomes to regulate stability/functional specificity and participates in development/stress adaptation.	[121]
Post-translational modification-associated factors	Kinases/phosphatases	It biases ribosomes toward stress-responsive gene translation.	[122]
	Methyltransferases	It regulates ribosome assembly/translation selectivity and participates in development/stress adaptation.	[84]
	Ubiquitin ligases/deubiquitinases	It maintains subunit homeostasis and participates in quality control/stress responses.	[99,100,101]

## Data Availability

No new data were created or analyzed in this study. Data sharing is not applicable to this article.
